# Genetic and Environmental Associations Among Pain, Sleep Disturbances, and Substance Use Intent in Early Adolescence

**DOI:** 10.1002/jad.70054

**Published:** 2025-09-21

**Authors:** Kit K. Elam, Angel Trevino, Jodi Kutzner, Jinni Su, Patrick D. Quinn

**Affiliations:** ^1^ Department of Applied Health Science Indiana University Bloomington Indiana USA; ^2^ Department of Psychology Arizona State University Tempe Arizona USA

**Keywords:** early adolescence, pain, sleep, substance use, twin

## Abstract

**Purpose:**

Pain, sleep disturbances, and substance use are common in adolescence, with research indicating that genetic and environmental factors account for variation in each of these behavioral and health outcomes. Moreover, pain, sleep disturbances, and substance use often co‐occur during adolescence. However, research has not examined whether there is genetic and/or environmental covariation across these constructs in early adolescence or in diverse samples. To address these gaps, we examined genetic and environmental covariation in pain, sleep disturbances, and substance use intent in early adolescence.

**Methods:**

Our study used data from the Adolescent Brain Cognitive Development (ABCD) twin subsample at age 12/13 (834 monozygotic twins (MZ) and 1262 dizygotic (DZ) twins; 50% female; 64% White, 14% African American, 12% Latinx/Hispanic, 10% multiracial/other). We estimated univariate twin ACE decompositions for the number of pain sites, sleep disturbances, and intent to use substances as well as pairwise bivariate Cholesky decompositions across phenotypes.

**Results:**

We found variation in pain attributable to additive genetic and unique environmental influences. Variation in sleep disturbances was due to additive genetic, unique environmental, and shared environmental influences. Variation in substance use intent was due to additive genetic and unique environmental influences. There was some evidence of shared genetic etiology across pain and sleep, and some evidence of shared environmental etiology across pain and substance use intent.

**Discussion:**

Findings highlight the importance of examining shared genetic and environmental etiology underlying pain, sleep, and substance use in early adolescence, which can help identify transdiagnostic targets for early prevention and interventions of health issues.

## 1 Introduction

Physical pain, sleep disturbances, and substance use are common in adolescence, and they often co‐occur. There is also evidence that biologic, genetic, and psychological factors contribute to each of these issues. Preliminary evidence in adult samples indicates there may be shared genetic and/or environmental etiology underlying comorbidity in these constructs, but this has not been examined in early adolescence or in racially/ethnically diverse samples. We address this in the current study by examining genetic and environmental covariation across pain, sleep disturbances, and substance use intent in early adolescence using the twin sample of the Adolescent Brain Cognitive Development (ABCD) study.

Estimates indicate 25%–40% of children and adolescents experience chronic or recurrent pain, that is, pain lasting longer than 3 months that interferes with functioning (Allen et al. 2016; King et al. 2011; Stanford et al. 2008). Moreover, risk for chronic pain increases across adolescence, with some evidence of higher incidence in girls compared to boys (Wager et al. 2020). When left untreated, chronic pain often persists into adulthood (Christensen et al. 2019). Similarly, during adolescence, 30%–40% of children experience sleep disturbances (e.g., poor quality sleep, difficulty falling asleep, or maintaining sleep), with high continuity in sleep issues across adolescence into adulthood (Fatima et al. 2016; Fricke‐Oerkermann et al. 2007; Gradisar et al. 2011). Chronic pain and sleep disturbances in childhood and adolescence often co‐occur and appear reciprocally related to one another. Adolescents with chronic pain experience poorer sleep quality and other disturbances because of pain symptoms, which in turn can exacerbate pain symptoms due to inadequate rest as well as increasing pain sensitivity (Christensen et al. 2019). These issues can be especially problematic in early adolescence as during this developmental period adolescents normatively experience more sleep deprivation while having increased needs for sleep (Palermo et al. 2008).

Sleep disturbances and pain in adolescence are also associated with substance use. Initiation of substance use is common in adolescence with recent estimates that 29% of adolescents use alcohol, 22% use marijuana, and ~15% have ever used an illicit substance excluding marijuana (Johnston et al. 2022; Jones 2020). Adolescent substance use is problematic for individual functioning and associated with impaired physical and mental health (Gray and Squeglia 2018). There is robust evidence that pain, sleep disturbances, and substance use are associated in adult samples (Herrero Babiloni et al. 2020; Basu et al. 2022; Compton and St. Marie 2022; Ditre et al. 2019). Accumulating evidence indicates that in adolescence, pain and sleep issues can lead to substance use. For instance, sleep disturbances in adolescence along with normative changes in circadian rhythms have been proposed to increase adolescents' susceptibility to substance use by increasing reward sensitivity (Brand and Kirov 2011; Gulick and Gamsby 2018; Gromov and Gromov 2009). Some studies also find that pain and substance use are associated in adolescence, possibly through attempts to cope with pain or related distress (Groenewald et al. 2019; Scaini et al. 2022). Genetic and environmental factors contribute to variation in pain, sleep, and substance use in adolescence but contributions to covariation have not been studied.

Traditional twin models can be used to estimate heritability for a behavior by decomposing variance into additive genetic (A), shared environmental (C), and unique environmental (E) components. Three recent reviews on the heritability of adolescent sleep find varying estimates for sleep duration (15%–69%), sleep quality (33%–44%), and insomnia and other specific sleep disturbances (62%–85%) (Kocevska et al. 2021; Lewis and Gregory 2021; Madrid‐Valero et al. 2020). There is some evidence that pain is weakly to moderately heritable in adolescence, (Battaglia et al. 2020; Champion et al. 2021) but findings bear replication given this limited literature. Substance use is also moderately heritability with estimates varying by substance type and age (Trucco and Hartmann 2021; Zellers et al. 2022). Some work has examined these constructs individually in ABCD. Estimates for the heritability of sleep functioning at age 9/10 in ABCD range from 0.20% to 0.80% (Maes et al. 2023). Overall pain severity, defined as the worst pain experienced over the last month on a 0–10 scale, was also found to be heritable at age 13 (0.12) but not at age 12 (Rader et al. 2023). This body of evidence suggests there are genetic and environmental contributions to pain, sleep, and substance use in adolescence. This has led to proposals that genetic and environmental covariation may contribute to pain, sleep disturbances, and substance use (Christensen et al. 2019).

In adult samples, twin studies have found genetic overlap in cannabis use and insomnia (Winiger et al. 2020). and cannabis use and sleep duration (Winiger et al. 2019). Molecular genetic studies in adults have found genetic correlations between substance use and sleep health (Hatoum et al. 2022), and among cannabis use, sleep deficits, and short sleep duration (Winiger et al. 2021, 2019). Using a Mendelian randomization approach, genetic correlations were found between chronic pain and multiple substance use disorders, supporting their causal effect (Koller et al. 2024), which is further supported by a review on genetic overlap between alcohol use disorder and chronic pain (Yeung et al. 2017). Only one related study exists in adolescence, which found genetic covariation in pain, anxiety, and affective problems in adolescence, and a latent factor across these constructs was phenotypically associated with substance use (Scaini et al. 2022). These findings have led researchers to propose that genetically influenced biological systems such as those involving dopamine, serotonin, and orexin may underlie commonality in pain sensitivity, sleep issues, and substance use (Christensen et al. 2019; Mun et al. 2024). Less research has focused on underlying environmental influences, but research has theorized that early distal factors such as trauma (Finan and Smith 2013) and environmental quality in childhood encompassing family SES, neighborhood disadvantage, and family environment may contribute to pain, sleep, and substance use (Valrie et al. 2013).

Using data from the twin sample of the ABCD Study, we extend this body of work by examining genetic and environmental covariation at age 12/13 among (1) youth report of pain via body map, (2) sleep disturbances, and (3) intent to use substances. Although evidence indicates there is genetic overlap in pain, sleep disturbances, and substance use in adult populations, this has yet to be examined in early adolescence prior to regular substance use when these issues typically emerge. Understanding shared etiology of these outcomes early in adolescence is important to inform the identification of transdiagnostic risk factors for early intervention efforts.

## 2 Methods

### 2.1 Participants

Data for this project were drawn from the ABCD Study (*N* = 11,875; 9–10 years old at baseline; 47.8% female; 52.1% non‐Hispanic White, 15.0% non‐Hispanic Black, 20.3% Hispanic/Latinx, 2.1% Asian, and 10.5% other [e.g., biracial]). The ABCD Study is an ongoing study following youth across the United States on their behavioral, social, and neurocognitive development from age 9/10 with planned assessments to age 18/19. In the current study, we used data from the age 12/13 assessment reflecting the developmental period of early adolescence. The analytic sample was based on available ABCD data which included a total of 2096 twins (417 monozygotic twin pairs (MZ) and 631 dizygotic (DZ) twin pairs; 50% female; 64% White, 14% African American, 12% Latinx/Hispanic, 10% multiracial/other).

### 2.2 Procedures

The ABCD Study recruited eligible children from a set of 21 nationally distributed study sites using probability sampling of schools within the study site areas, with < 10% of the final sample recruited using alternative procedures such as mailing lists, referrals, and twin registries (Garavan et al. 2018). A twin subsample was recruited through four twin‐research sites. Twins were identified through state birth records at each study site. As part of the ABCD Study, parents provided consent for their participation and written informed consent for youth participants. Youths provided their assent. Baseline data collection occurred between September 1, 2016 and November 15, 2018. The ABCD Study includes neurocognitive, mental and physical health assessments, environmental and cultural measures, structural and functional brain imaging, as well as whole genome genotyping. Data are shared with the research community through annual data releases via the NIMH Data Archive. Data used in the present study came from ABCD data release 4.0. As part of the ABCD Study, parents and children are assessed annually, with smaller assessments occurring midyear. The baseline assessment occurred when youth were 9/10 years old, with annual assessments occurring at the 1‐year follow‐up (youth aged 10/11), the 2‐year follow‐up (youth aged 11/12), and the 3‐year follow‐up (youth aged 12/13). This study was approved by the Indiana University Institutional Review Board. ABCD data are publicly available from the NIMH National Data Archive (https://nda.nih.gov/) and analysis code for the current study is available upon request.

### 2.3 Measures

#### 2.3.1 Pain

Youth completed the Collaborative Health Outcomes Information Registry electronic body map (Scherrer et al. 2021). This measure assesses the presence of pain in anterior and posterior body sites (yes/no). Youth were presented with a male or female body map and directed to “Please touch each body region where you have felt pain during the last month.” The most common pain sites for boys were the front of the right knee (2.9%) and front of left knee (2.8%). The most common pain sites for girls were the front right of the abdomen (4.4%) and front left of the abdomen (4.6%). The total number of body sites was collapsed across males and females and categorized as follows (0 = 0 pain sites, 1 = 1 pain site, 2 = 2 pain sites, 3 = 3–5 pain sites, 4 = > 5 pain sites). This coding yielded ~5% or greater of the sample for each endorsement category (0 = 68%, 1 = 5%, 2 = 10%, 3 = 8%, 4 = 9%).

#### 2.3.2 Sleep Disturbances

Parents completed the Sleep Disturbance Scale for Children (SDSC) (Bruni et al. 1996). This 26‐item measure assesses sleep problems on a 5‐point scale in six areas: (1) arousal or nightmares, (2) initiating or maintaining sleep, (3) excessive sleep, (4) sleep breathing disorders, (5) sleep hyperhidrosis, (6) sleep‐wake transition. The six subscales were first computed then the subscales were averaged together to form an overall measure of average sleep issues. Thus, higher scores reflect greater average sleep disturbances (Cronbach's *α *= 0.82).

#### 2.3.3 Substance Use Intent

At age 12–13, youth reported on six items assessing their intent to use substances and curiosity about various substances (i.e., alcohol and marijuana) based on items modified from the Population Assessment of Tobacco and Health study (Hyland et al. 2017). Items were modified to reflect alcohol and marijuana and captured youth's curiosity (“Have you ever been curious about drinking/trying alcohol/marijuana” 1* = very curious,* 4* = not curious at all*), intent to use (“Do you think you will try alcohol/marijuana soon?” 1* = definitely yes,* 4 *= definitely not*), and proclivity to use with peers (“If one of your best friends were to offer you alcohol/marijuana, would you try it?” 1 *= definitely yes,* 4 *= definitely not*). Items were reverse coded to reflect greater intent to use substances (Cronbach's *α* = 0.84). The measure of substance use intent was highly kurtotic due to low endorsement of substance use intent so final scores were dichotomized to capture no substance use intent/curiosity (0) which encompassed responses of *not curious at all* and *definitely not*, and any substance use intent/curiosity (1) which encompassed all other responses.

### 2.4 Analytic Approach

As part of preliminary analysis in SPSS v28, we examined mean levels and correlations among pain, sleep disturbances, and substance use intent in the twin subsample for key variables. This was followed by cross‐twin correlations separately for MZ and DZ twins.

We first examined genetic and environmental variation in each phenotype by conducting univariate twin ACE models in Mplus. This approach decomposes variance within a phenotype into three subcomponents: additive genetic effects (A), common/shared environmental effects (C), and nonshared environmental effects (E). The best‐fitting model was chosen based on comparisons of a series of nested models (comparing ACE, AE, CE, and E models). Within univariate models, pain and sleep were treated as continuous variables and substance use intent was treated as a categorical (dichotomous) variable using a Theta parameterization (Loehlin 1996; Prescott 2004).

To examine genetic and environment covariation across phenotypes, we conducted bivariate Cholesky decompositions in Mplus (Loehlin 1996; Prescott 2004). These models estimate to what extent genetic and environmental influences on one behavior (e.g., pain) are correlated with another behavior (e.g., sleep), as well as unique and shared variation in A, C, and E parameters (Loehlin 1996; Prescott 2004). We first estimated full bivariate models including all ACE parameters for both phenotypes. We followed this by examining reduced bivariate models in which we dropped A, C, or E parameters for one or both phenotypes where indicated by the absence of effects in the univariate models and bivariate model fit. Age and sex were covaried in all models. We computed 95% confidence intervals using bootstrapping (1000 resamples) for all models.

## 3 Results

Descriptive statistics can be found in Table 1. There were low levels of pain, with the majority of youth reporting no pain sites and 32% of youth reporting one or more pain sites. Average sleep disturbances were also low although 97% of parents reported their child had at least one sleep disturbance symptom. In total, 30% of youth reported some curiosity or intent to use substances in the near future. In the full twin sample, pain was weakly correlated with sleep disturbances and substance use intent. Correlations across MZ twins indicated associations within construct but not across constructs. In DZ twins, there was a cross‐twin association within sleep disturbances.

**Table 1:** Descriptive statistics.

	Pain	Sleep disturbances	Substance use intent
M/SD or percent	32%	1.29 (0.24)	30%
Phenotypic correlations			
Pain	—		
Sleep disturbances	**0.14 (0.10, 0.19)**	—	
Substance use intent	**0.08 (0.03, 0.13)**	0.03 (−0.02, 0.08)	—
MZ correlations across twin			
Pain	**0.21 (0.10, 0.32)**		
Sleep disturbances	−0.01 (−0.05, 0.04)	**0.64 (0.49, 0.79)**	
Substance use intent	−0.06 (−0.13, 0.01)	0.01 (−0.05, 0.08)	**0.26 (0.12, 0.40)**
DZ correlations across twin			
Pain	0.07 (−0.02, 0.16)		
Sleep disturbances	0.04 (−0.03, 0.10)	**0.45 (0.37, 0.55)**	
Substance use intent	0.06 (−0.01, 0.11)	0.02 (−0.04, 0.07)	0.08 (−0.02, 0.18)

*Note:* Significant effects are bolded.

Results from univariate ACE models can be found in Table 2. We interpreted the AE model as having the best fit for pain, compared to the CE model, based on the lack of shared environmental variance in the full ACE model. In the AE model, unique environmental influences explained the greatest proportion of variance in pain (*e* ^2^ = 0.82, 95% CI: [0.70, 0.89]) with a small proportion of variance explained by additive genetic influences (*a* ^2^ = 0.18, 95% CI: [0.05, 0.33]). The full ACE model fit best for sleep disturbances. Additive genetic influences explained approximately half the variance (*a* ^2^ = 0.53, 95% CI: [0.31, 0.75]), with smaller proportions accounted for by shared (*c* ^2^ = 0.19, 95% CI: [0.01, 0.37]) and unique (*e* ^2^ = 0.28, 95% CI: [0.21, 0.37]) environmental influences. The AE model fit best for substance use intent, and estimates were similar to the full ACE model. Variance in substance use intent was accounted for by small additive genetic influences (*a* ^2^ = 0.21, 95% CI: [0.03, 0.31]) with a large proportion accounted for by unique environmental influences (*e* ^2^ = 0.78, 95% CI: [0.69, 0.90]).

**Table 2:** Univariate ACE models adjusted for covariates.

	A			C			E		AIC		−2LL		df	Δ −2LL		Δ df		*p*
Pain sites																		
ACE	0.18			0			0.82		5351.75		5343.74		6	NA		NA		NA
**AE**	**0.18**			—			**0.82**		**5349.75**		**5343.74**		**7**	**0**		**1**		**1.00**
CE	—			0.13			0.86		5352.72		5346.15		7	2.41		1		0.12
E	—			—			1		5371.27		5361.28		8	17.54		2		< 0.001
Sleep disturbances																		
**ACE**	**0.53**			**0.19**			**0.28**		**4877.24**		**4869.24**		**6**	**NA**		**NA**		**NA**
AE	0.74			—			0.27		4882.58		4876.58		7	7.34		1		0.007
CE	—			0.55			0.45		4922.75		4916.76		7	47.52		1		< 0.001
E	—			—			1		3635.16		5251.93		8	382.69		2		< 0.001
Substance use intent																		
ACE	0.21			0			0.78		5116.21		5108.22		6	NA		NA		NA
**AE**	**0.21**			**—**			**0.78**		**5114.21**		**5108.22**		**7**	**0**		**1**		**1.0**
CE	—			0.15			0.85		5118.31		5112.32		7	4.10		1		0.04
E	—			—			1		5141.54		5131.54		8	23.32		2		< 0.001

*Note:* A, C, and E are standardized additive genetic, shared environmental, nonshared environmental variance components. Bolded line indicates best‐fitting model.

Abbreviations: −2LL = −2 log likelihood, AIC = Akaike's Information Criterion, ∆ = change.

Genetic and environmental correlations from the bivariate ACE models with Cholesky decomposition are in Table 3. There was no evidence of shared genetic or unique environmental variance across pain and sleep with a fully saturated estimate for the shared environmental correlation (rC = 1.00, 95% CI: [1.00, 1.00]). Based on this and univariate model results, we examined the reduced AE model for pain within the bivariate model, which had the same fit (ΔAIC = 0.0; Δ*χ* ^2^ = 0.0). In the reduced model, we found that dropping the C path for pain led to a significant genetic correlation (rA = 0.41, 95% CI: [0.19, 0.68]). In the full pain and substance use intent model a small unique environmental correlation was detected (rE = 0.09, 95% CI: [0.02, 0.17]), but there was no genetic association and a fully saturated estimate was returned for shared environmental influence (rC = 1.00, 95% CI: [1.00, 1.00]). When testing the reduced AE models for both pain and substance use intent in the bivariate model, there was a similar fit to the full model (ΔAIC = 4.25; Δ*χ* ^2^ = 1.751). In the reduced model, dropping C paths for pain and substance use attenuated the unique environmental correlation. No significant associations were detected in either the full or reduced model for sleep and substance use intent. Unique and shared variance estimates from full bivariate models can be found in Supplemental Table S1.

**Table 3:** Genetic (rA), shared environmental (rC), and unique environmental (rE) correlations across phenotypes.

	rA estimate (95% CI)	rC estimate (95% CI)	rE estimate (95% CI)
Pain‐Sleep full model	0.09 (−1.00, 0.09)	1.00 (1.00, 1.00)	0.06 (−0.04, 0.15)
Pain‐Sleep reduced model	**0.41 (0.19, 0.68)**	—	0.03 (−0.07, 0.12)
Pain‐Intent full model	−0.59 (−1.00, 0.31)	1.00 (1.00, 1.00)	**0.09 (0.02, 0.17)**
Pain‐Intent reduced model	0.10 (−0.23, 0.39)	—	0.07 (−0.01, 0.15)
Sleep‐Intent full model	−0.05 (−1.00, 0.55)	1.00 (1.00, 1.00)	0.003 (−0.13, 0.13)
Sleep‐Intent reduced model	−0.05 (−1.00, 0.48)	—	0.003 (−0.13, 0.13)

*Note:* Bolded estimates indicate significance based on 95% Confidence Interval.

## 4 Discussion

The current study examined genetic and environmental covariation across pain, sleep disturbances, and substance use intent in early adolescence. This extends existing literature in adults by investigating shared genetic and environmental factors underlying these important health issues at an earlier developmental stage. Importantly, examining genetic and environmental covariation can help in identifying conceptual risk factors, guiding future research and applied endeavors addressing these behaviors.

In univariate ACE models, we found substance use to be largely accounted for by unique environmental factors whereas sleep disturbances were accounted for by genetic and environmental factors. Our findings on sleep and substance use partially align with previous research in adolescence finding both genetic and environmental variation in sleep quality, quantity, disturbances, and for various substances (Kocevska et al. 2021; Lewis and Gregory 2021; Madrid‐Valero et al. 2020; Trucco and Hartmann 2021; Zellers et al. 2022; Maes et al. 2023; Breitenstein et al. 2021). However, the small genetic estimates found for substance use intent diverges from some previous research finding moderate genetic influences on substance use which may be a result of examining *intent* to use substances prior to regular substance use rather than actual substance use.

With regards to pain, we found variation in pain sites largely due to environmental factors which aligns with previous research (Battaglia et al. 2020; Champion et al. 2021). Accumulating evidence appears to indicate that heritability in pain increases later in life, such as during early to mid‐adulthood (Mogil 2021). More broadly, behavioral genetic research finds that genetic influences for most complex behaviors increase from adolescence into adulthood (Bergen et al. 2007). Theoretical perspectives propose this is due to the emergence of new genetic influences and genetic interplay with social processes across development (Reiss and Neiderhiser 2000; Elam et al. 2023). It is also possible that genetic influences on pain increase over time due to increases in the prevalence of pain, the emergence of chronic pain, and more accurate reporting of pain with age. Lastly, our measure of pain, number of pain sites, represents a single aspect of experiencing pain which is likely distinct from chronicity and/or intensity of pain. Future research in youth should examine the genetic and environmental bases of various pain measures. The present study extends previous univariate twin research to early adolescence in a nationally distributed sample of racially/ethnically diverse youth using youth‐reported pain and examination of intent to use substances prior to regular substance use onset.

The reduced bivariate model indicated there were genetically driven associations among pain and sleep. Pain and sleep disturbances in childhood and adolescence often co‐occur and are reciprocally related to one another. There is some corresponding research in adults that finds genetic correlations between low back pain and sleep quality (Pinheiro et al. 2018) as well as sleep quality and pain. Gasperi et al. (2017). It may be that there are common neurobiological pathways underlying pain and sleep quality in early adolescence, such as in dopamine or orexin systems, which may increase pain sensitivity and disrupt sleep/wake patterns (Sivertsen et al. 2015; Monti and Monti 2007).

Conversely, the reduced bivariate models for pain and substance use intent as well as sleep and substance use intent did not find any genetic covariation. As previously mentioned, this may be because there were very small genetic influences on substance use intent and pain in early adolescence in our univariate models. As genetic influences grow over time it may be that genetic correlations across pain, sleep, and substance use only emerge in later adolescence or adulthood. For example, previous literature in adult samples finds moderate genetic correlations and relatively smaller environmental overlap between pain and substance use (Winiger et al. 2020, 2019). The lack of genetic associations may also be because of the low prevalence of intent to use substances in this developmental period or because intent to use substances has a different genetic basis than actual substance use.

In the full bivariate model for pain and substance use intent there was some evidence of a weak environmental association, although this was attenuated and no longer statistically significant in the reduced model. It could be that environmental determinants partially underlie the co‐occurrence of pain and substance use intent, however, very little research has examined social or cultural factors underlying pain and substance use in adolescence. One study found poor family functioning associated with lower back pain and cigarette use in early adolescence (Gill et al. 2014). This is supported by theoretical models positing that in adolescence family and peer risk factors can contribute to pain and opioid use (Dash et al. 2018), and more generally that sociodemographic factors such as low SES and racial/ethnic minority (Ditre et al. 2019) status are transdiagnostic risk factors. Collectively, there is a marked lack of research on genetic and sociocultural risk and protective factors underlying pain, sleep, and substance use in adolescence compared to a relative emphasis on biological mechanisms (Herrero Babiloni et al. 2020).

It is important to note that there were no significant cross‐trait cross‐twin correlations which can indicate difficulty in estimating bivariate Cholesky models. However, we conducted the aforementioned bivariate models as we believe it is important to examine for shared genetic and/or environmental contributions even if effects are small in size. This approach allows us to determine the extent to which effects are shared and/or unique, or where there are null associations. These findings, including the absence of associations, can be useful in understanding theoretical relationships between our constructs as well as informing future research. Relatedly, we note that the presence of fully saturated rC estimates in the bivariate Cholesky models may suggest overfit models. To address this, we conducted reduced models without the common shared environmental path which we interpreted as the most accurate. It may be that there is little shared environmental variation in common between pain and substance use intent cross‐sectionally during this developmental period, especially prior to more widespread chronic pain and the onset of substance use in adolescence.

Limitations of our study include a relatively small twin sample that precluded examination of variation by gender and race/ethnicity. This also may have contributed to relatively weak cross‐trait cross‐twin correlations which a larger sample could possibly better detect. Also, current rates of substance use are very low in ABCD given participants' young age, as well as decreasing national trends in adolescent alcohol and marijuana use. Finally, effect sizes were very small in bivariate models. Our examination of substance use intent was a strength but may differ from findings for actual substance use which may have different genetic and environmental bases.

In light of our findings and relatively small effect sizes, the current findings emphasize the need for research using larger samples of twin youth to study pain, sleep, and substance use behaviors. Also, given the reciprocal nature of pain, sleep, and substance use behaviors over time a future direction for research is examining associations in longitudinal models using the current ABCD data at age 12/13 and older ages as they become available. This has potential to identify transdiagnostic genetic risk factors for pain and sleep, plus precursors of substance use in early adolescence. The prevention of pain, sleep, and substance use issues early in life is of vital importance given evidence that these issues exacerbate one another over time (Battaglia et al. 2023; Palermo 2020), and can serve as risk factors for mental health and substance use disorders, including opioid use, as well as physical health issues later in life. Current transdiagnostic efforts typically focus on biological interventions, such as Orexin agonists (Mun et al. 2024) and individual‐level psychosocial risk factors, such as negative affect, anxiety, and depression (Ditre et al. 2019). Although not strongly indicated by our findings, concurrent identification of social, sociodemographic, and cultural risk factors in diverse populations would complement these lines of research and ensure equitable benefit of research findings.

## 5 Implications and Contributions

This study advances research on pain, sleep, and substance use behaviors by studying shared underlying influences in a diverse early adolescent sample. In early adolescence, there was some evidence that pain and sleep share an underlying genetic influence and some evidence that pain and intent to use substances share an underlying environmental influence. These findings bear replication in larger sample sizes and across early adolescence.

## Author Contributions

Kit K. Elam contributed to the conceptualization, methodology, formal analysis, writing and revision of the manuscript. Angel Trevino contributed to the data curation, methodology, and writing of the manuscript. Jodi Kutzner contributed to the data curation, methodology, writing and revision of the manuscript. Jinni Su contributed to the conceptualization, methodology, and writing of the manuscript. Patrick D. Quinn contributed to the conceptualization, methodology, writing and revision of the manuscript.

## Ethics Statement

This study was approved by the Indiana University Institutional Review Board (#2008226356).

## Conflicts of Interest

The authors declare no conflicts of interest.

## Supporting information


**Supplemental Table 1:** ACE Estimates and Unique and Shared Variance Estimates from Full Bivariate Models.

## Data Availability

ABCD data are publicly available from the NIMH National Data Archive (https://nda.nih.gov/) and the analysis code for the current study is available upon request. This study was not preregistered.

## References

[jad70054-bib-0001] Allen, J. M. , D. M. Graef , J. H. Ehrentraut , B. L. Tynes , and V. M. Crabtree . 2016. “Sleep and Pain in Pediatric Illness: A Conceptual Review.” CNS Neuroscience & Therapeutics 22, no. 11: 880–893. 27421251 10.1111/cns.12583 PMC6492850

[jad70054-bib-0002] Basu, A. , N. Anand , and M. Das . 2022. “Sleep and Substance‐Use Disorder.” In Sleep and Neuropsychiatric Disorders, 435–464. Springer.

[jad70054-bib-0003] Battaglia, M. , G. Garon‐Carrier , M. Brendgen , et al. 2020. “Trajectories of Pain and Anxiety in a Longitudinal Cohort of Adolescent Twins.” Depression and Anxiety 37, no. 5: 475–484. 31944483 10.1002/da.22992

[jad70054-bib-0004] Battaglia, M. , C. B. Groenewald , F. Campbell , et al. 2023. “We Need to Talk: The Urgent Conversation on Chronic Pain, Mental Health, Prescribing Patterns and the Opioid Crisis.” Journal of Psychopharmacology 37, no. 5: 437–448. 37171242 10.1177/02698811221144635

[jad70054-bib-0005] Bergen, S. E. , C. O. Gardner , and K. S. Kendler . 2007. “Age‐Related Changes in Heritability of Behavioral Phenotypes Over Adolescence and Young Adulthood: A Meta‐Analysis.” Twin Research and Human Genetics 10, no. 3: 423–433. 17564500 10.1375/twin.10.3.423

[jad70054-bib-0006] Brand, S. , and R. Kirov . 2011. “Sleep and Its Importance in Adolescence and in Common Adolescent Somatic and Psychiatric Conditions.” International Journal of General Medicine 4: 425–442. 21731894 10.2147/IJGM.S11557 PMC3119585

[jad70054-bib-0007] Breitenstein, R. S. , L. D. Doane , and K. Lemery‐Chalfant . 2021. “Children's Objective Sleep Assessed With Wrist‐Based Accelerometers: Strong Heritability of Objective Quantity and Quality Unique From Parent‐Reported Sleep.” Sleep 44, no. 1: zsaa142. 32729904 10.1093/sleep/zsaa142 PMC7819846

[jad70054-bib-0008] Bruni, O. , S. Ottaviano , V. Guidetti , et al. 1996. “The Sleep Disturbance Scale for Children (SDSC) Construct Ion and Validation of an Instrument to Evaluate Sleep Disturbances in Childhood and Adolescence.” Journal of Sleep Research 5, no. 4: 251–261. 9065877 10.1111/j.1365-2869.1996.00251.x

[jad70054-bib-0009] Champion, D. , M. Bui , A. Bott , et al. 2021. “Familial and Genetic Influences on the Common Pediatric Primary Pain Disorders: A Twin Family Study.” Children 8, no. 2: 89. 33525537 10.3390/children8020089 PMC7911833

[jad70054-bib-0010] Christensen, J. , M. Noel , and R. Mychasiuk . 2019. “Neurobiological Mechanisms Underlying the Sleep‐Pain Relationship in Adolescence: A Review.” Neuroscience and Biobehavioral Reviews 96: 401–413. 30621863 10.1016/j.neubiorev.2018.11.006

[jad70054-bib-0011] Compton, P. , and B. St. Marie . 2022. “Coexisting Substance Use Disorder and Chronic Pain During COVID‐19.” Pain Management Nursing 23, no. 1: 17–25. 34620549 10.1016/j.pmn.2021.08.011 PMC8418911

[jad70054-bib-0012] Dash, G. F. , A. C. Wilson , B. J. Morasco , and S. W. Feldstein Ewing . 2018. “A Model of the Intersection of Pain and Opioid Misuse in Children and Adolescents.” Clinical Psychological Science 6, no. 5: 629–646. 30333942 10.1177/2167702618773323 PMC6186448

[jad70054-bib-0013] Ditre, J. W. , E. L. Zale , and L. R. LaRowe . 2019. “A Reciprocal Model of Pain and Substance Use: Transdiagnostic Considerations, Clinical Implications, and Future Directions.” Annual Review of Clinical Psychology 15, no. 1: 503–528. 10.1146/annurev-clinpsy-050718-095440 30566371

[jad70054-bib-0014] Elam, K. K. , K. Lemery‐Chalfant , and L. Chassin . 2023. “A Gene‐Environment Cascade Theoretical Framework of Developmental Psychopathology.” Journal of Psychopathology and Clinical Science 132, no. 3: 287–296. 36201798 10.1037/abn0000546 PMC10076453

[jad70054-bib-0015] Fatima, Y. , S. A. R. Doi , M. O'Callaghan , G. Williams , J. M. Najman , and A. A. Mamun . 2016. “Parent and Adolescent Reports in Assessing Adolescent Sleep Problems: Results From a Large Population Study.” Acta Paediatrica 105, no. 9: e433–e439. 26991850 10.1111/apa.13404

[jad70054-bib-0016] Finan, P. H. , and M. T. Smith . 2013. “The Comorbidity of Insomnia, Chronic Pain, and Depression: Dopamine as a Putative Mechanism.” Sleep Medicine Reviews 17, no. 3: 173–183. 22748562 10.1016/j.smrv.2012.03.003 PMC3519938

[jad70054-bib-0017] Fricke‐Oerkermann, L. , J. Plück , M. Schredl , et al. 2007. “Prevalence and Course of Sleep Problems in Childhood.” Sleep 30, no. 10: 1371–1377. 17969471 10.1093/sleep/30.10.1371 PMC2266270

[jad70054-bib-0018] Garavan, H. , H. Bartsch , K. Conway , et al. 2018. “Recruiting the ABCD Sample: Design Considerations and Procedures.” Developmental Cognitive Neuroscience 32: 16–22. 29703560 10.1016/j.dcn.2018.04.004 PMC6314286

[jad70054-bib-0019] Gasperi, M. , M. Herbert , E. Schur , D. Buchwald , and N. Afari . 2017. “Genetic and Environmental Influences on Sleep, Pain, and Depression Symptoms in a Community Sample of Twins.” Psychosomatic Medicine 79, no. 6: 646–654. 28658193 10.1097/PSY.0000000000000456 PMC5501256

[jad70054-bib-0020] Gill, D. K. , M. C. Davis , A. J. Smith , and L. M. Straker . 2014. “Bidirectional Relationships Between Cigarette Use and Spinal Pain in Adolescents Accounting for Psychosocial Functioning.” British Journal of Health Psychology 19, no. 1: 113–131. 23552050 10.1111/bjhp.12039

[jad70054-bib-0021] Gradisar, M. , G. Gardner , and H. Dohnt . 2011. “Recent Worldwide Sleep Patterns and Problems During Adolescence: A Review and Meta‐Analysis of Age, Region, and Sleep.” Sleep Medicine 12, no. 2: 110–118. 21257344 10.1016/j.sleep.2010.11.008

[jad70054-bib-0022] Gray, K. M. , and L. M. Squeglia . 2018. “Research Review: What Have We Learned About Adolescent Substance Use?” Journal of Child Psychology and Psychiatry 59, no. 6: 618–627. 28714184 10.1111/jcpp.12783 PMC5771977

[jad70054-bib-0023] Groenewald, C. B. , E. F. Law , E. Fisher , S. E. Beals‐Erickson , and T. M. Palermo . 2019. “Associations Between Adolescent Chronic Pain and Prescription Opioid Misuse in Adulthood.” Journal of Pain 20, no. 1: 28–37. 30098405 10.1016/j.jpain.2018.07.007 PMC6309740

[jad70054-bib-0024] Gromov, I. , and D. Gromov . 2009. “Sleep and Substance Use and Abuse in Adolescents.” Child and Adolescent Psychiatric Clinics of North America 18, no. 4: 929–946. 19836697 10.1016/j.chc.2009.04.004

[jad70054-bib-0025] Gulick, D. , and J. J. Gamsby . 2018. “Racing the Clock: The Role of Circadian Rhythmicity in Addiction Across the Lifespan.” Pharmacology & Therapeutics 188: 124–139. 29551440 10.1016/j.pharmthera.2018.03.003

[jad70054-bib-0026] Hatoum, A. S. , E. A. Winiger , C. L. Morrison , E. C. Johnson , and A. Agrawal . 2022. “Characterisation of the Genetic Relationship Between the Domains of Sleep and Circadian‐Related Behaviours With Substance Use Phenotypes.” Addiction Biology 27, no. 4: e13184. 35754104 10.1111/adb.13184 PMC10038127

[jad70054-bib-0027] Herrero Babiloni, A. , B. P. De Koninck , G. Beetz , L. De Beaumont , M. O. Martel , and G. J. Lavigne . 2020. “Sleep and Pain: Recent Insights, Mechanisms, and Future Directions in the Investigation of This Relationship.” Journal of Neural Transmission 127: 647–660. 31452048 10.1007/s00702-019-02067-z

[jad70054-bib-0028] Hyland, A. , B. K. Ambrose , K. P. Conway , et al. 2017. “Design and Methods of the Population Assessment of Tobacco and Health (PATH) Study.” Tobacco Control 26, no. 4: 371–378. 27507901 10.1136/tobaccocontrol-2016-052934 PMC5299069

[jad70054-bib-0029] Johnston, L. D. , R. A. Miech , P. M. O'Malley , J. G. Bachman , J. E. Schulenberg , and M. E. Patrick . 2022. *Monitoring the Future National Survey Results on Drug Use, 1975‐2021: Overview, Key Findings on Adolescent Drug Use*. Institute for Social Research, University of Michigan.

[jad70054-bib-0030] Jones, C. M. H. B. Clayton , N. P. Deputy , et al. 2020. “Prescription Opioid Misuse and Use of Alcohol and Other Substances Among High School Students—Youth Risk Behavior Survey, United States, 2019.” MMWR Supplements 69: 38–46. 32817608 10.15585/mmwr.su6901a5 PMC7440199

[jad70054-bib-0031] King, S. , C. T. Chambers , A. Huguet , et al. 2011. “The Epidemiology of Chronic Pain in Children and Adolescents Revisited: A Systematic Review.” Pain 152, no. 12: 2729–2738. 22078064 10.1016/j.pain.2011.07.016

[jad70054-bib-0032] Kocevska, D. , N. L. Barclay , W. M. Bramer , P. R. Gehrman , and E. J. W. Van Someren . 2021. “Heritability of Sleep Duration and Quality: A Systematic Review and Meta‐Analysis.” Sleep Medicine Reviews 59: 101448. 33636423 10.1016/j.smrv.2021.101448

[jad70054-bib-0033] Koller, D. , E. Friligkou , B. Stiltner , et al. 2024. “Pleiotropy and Genetically Inferred Causality Linking Multisite Chronic Pain to Substance Use Disorders.” Molecular Psychiatry 29: 2021–2030. 38355787 10.1038/s41380-024-02446-3 PMC11324857

[jad70054-bib-0034] Lewis, K. J. , and A. M. Gregory . 2021. “Heritability of Sleep and Its Disorders in Childhood and Adolescence.” Current Sleep Medicine Reports 7: 155–166. 34840933 10.1007/s40675-021-00216-z PMC8607788

[jad70054-bib-0035] Loehlin, J. C. 1996. “The Cholesky Approach: A Cautionary Note.” Behavior Genetics 26: 65–69.

[jad70054-bib-0036] Madrid‐Valero, J. J. , M. Rubio‐Aparicio , A. M. Gregory , J. Sánchez‐Meca , and J. R. Ordoñana . 2020. “Twin Studies of Subjective Sleep Quality and Sleep Duration, and Their Behavioral Correlates: Systematic Review and Meta‐Analysis of Heritability Estimates.” Neuroscience and Biobehavioral Reviews 109: 78–89. 31899301 10.1016/j.neubiorev.2019.12.028

[jad70054-bib-0037] Maes, H. H. M. , D. M. Lapato , J. E. Schmitt , et al. 2023. “Genetic and Environmental Variation in Continuous Phenotypes in the ABCD Study®.” Behavior Genetics 53, no. 1: 1–24. 36357558 10.1007/s10519-022-10123-w PMC9823057

[jad70054-bib-0038] Mogil, J. S. 2021. “Sources of Individual Differences in Pain.” Annual Review of Neuroscience 44, no. 1: 1–25. 10.1146/annurev-neuro-092820-105941 34236890

[jad70054-bib-0039] Monti, J. M. , and D. Monti . 2007. “The Involvement of Dopamine in the Modulation of Sleep and Waking.” Sleep Medicine Reviews 11, no. 2: 113–133. 17275369 10.1016/j.smrv.2006.08.003

[jad70054-bib-0040] Mun, C. J. , M. J. Reid , S. Sarandos , K. K. Elam , C. Li , and J. C. Strickland . 2024. “Improving Sleep to Address Co‐Occurring Substance Use Disorder and Chronic Pain: Exploring the Potential of the Orexin (Hypocretin) System as a Clinical Target. *Current Addiction Reports* 11, no. 6: 952–964.

[jad70054-bib-0041] Palermo, T. M. 2020. “Pain Prevention and Management Must Begin in Childhood: The Key Role of Psychological Interventions.” Pain 161: S114–S121. 33090744 10.1097/j.pain.0000000000001862 PMC7566813

[jad70054-bib-0042] Palermo, T. M. , I. Fonareva , and N. R. Janosy . 2008. “Sleep Quality and Efficiency in Adolescents With Chronic Pain: Relationship With Activity Limitations and Health‐Related Quality of Life.” Behavioral Sleep Medicine 6, no. 4: 234–250. 18853307 10.1080/15402000802371353 PMC3163674

[jad70054-bib-0043] Pinheiro, M. B. , J. J. Morosoli , M. L. Ferreira , et al. 2018. “Genetic and Environmental Contributions to Sleep Quality and Low Back Pain: A Population‐Based Twin Study.” Psychosomatic Medicine 80, no. 3: 263–270. 29240646 10.1097/PSY.0000000000000548

[jad70054-bib-0044] Prescott, C. A. 2004. “Using the Mplus Computer Program to Estimate Models for Continuous and Categorical Data From Twins.” Behavior Genetics 34: 17–40. 14739694 10.1023/B:BEGE.0000009474.97649.2f

[jad70054-bib-0045] Rader, L. , S. M. Freis , and N. P. Friedman . 2023. “Associations Between Adolescent Pain and Psychopathology in the Adolescent Brain Cognitive Development (ABCD) Study.” Behavior Genetics 53, no. 3: 232–248. 37036551 10.1007/s10519-023-10138-x PMC10246734

[jad70054-bib-0046] Reiss, D. , and J. M. Neiderhiser . 2000. “The Interplay of Genetic Influences and Social Processes in Developmental Theory: Specific Mechanisms Are Coming Into View.” Development and Psychopathology 12, no. 3: 357–374. 11014743 10.1017/s0954579400003060

[jad70054-bib-0047] Scaini, S. , G. Michelini , S. De Francesco , et al. 2022. “Adolescent Pain, Anxiety, and Depressive Problems: A Twin Study of Their Co‐Occurrence and the Relationship to Substance Use.” Pain 163, no. 3: e488–e494. 34294665 10.1097/j.pain.0000000000002400

[jad70054-bib-0048] Scherrer, K. H. , M. S. Ziadni , J.‐T. Kong , et al. 2021. “Development and Validation of the Collaborative Health Outcomes Information Registry Body Map.” Pain Reports 6, no. 1: e880. 33490848 10.1097/PR9.0000000000000880 PMC7813550

[jad70054-bib-0049] Sivertsen, B. , T. Lallukka , K. J. Petrie , Ó. A. Steingrímsdóttir , A. Stubhaug , and C. S. Nielsen . 2015. “Sleep and Pain Sensitivity in Adults.” Pain 156, no. 8: 1433–1439. 25915149 10.1097/j.pain.0000000000000131

[jad70054-bib-0050] Stanford, E. A. , C. T. Chambers , J. C. Biesanz , and E. Chen . 2008. “The Frequency, Trajectories and Predictors of Adolescent Recurrent Pain: A Population‐Based Approach.” Pain 138, no. 1: 11–21. 18093737 10.1016/j.pain.2007.10.032

[jad70054-bib-0051] Trucco, E. M. , and S. A. Hartmann . 2021. “Understanding the Etiology of Adolescent Substance Use Through Developmental Perspectives.” Child Development Perspectives 15, no. 4: 257–264. 34987602 10.1111/cdep.12426 PMC8722499

[jad70054-bib-0052] Valrie, C. R. , M. H. Bromberg , T. Palermo , and L. E. Schanberg . 2013. “A Systematic Review of Sleep in Pediatric Pain Populations.” Journal of Developmental and Behavioral Pediatrics 34, no. 2: 120–128. 23369958 10.1097/DBP.0b013e31827d5848 PMC3562475

[jad70054-bib-0053] Wager, J. , D. Brown , A. Kupitz , N. Rosenthal , and B. Zernikow . 2020. “Prevalence and Associated Psychosocial and Health Factors of Chronic Pain in Adolescents: Differences by Sex and Age.” European Journal of Pain 24, no. 4: 761–772. 31889351 10.1002/ejp.1526

[jad70054-bib-0054] Winiger, E. A. , J. M. Ellingson , C. L. Morrison , et al. 2021. “Sleep Deficits and Cannabis Use Behaviors: An Analysis of Shared Genetics Using Linkage Disequilibrium Score Regression and Polygenic Risk Prediction.” Sleep 44, no. 3: zsaa188. 32935850 10.1093/sleep/zsaa188 PMC7953210

[jad70054-bib-0055] Winiger, E. A. , S. B. Huggett , A. S. Hatoum , et al. 2020. “Onset of Regular Cannabis Use and Young Adult Insomnia: An Analysis of Shared Genetic Liability.” Sleep 43, no. 5: zsz293. 31855253 10.1093/sleep/zsz293 PMC7368342

[jad70054-bib-0056] Winiger, E. A. , S. B. Huggett , A. S. Hatoum , M. C. Stallings , and J. K. Hewitt . 2019. “Onset of Regular Cannabis Use and Adult Sleep Duration: Genetic Variation and the Implications of a Predictive Relationship.” Drug and Alcohol Dependence 204: 107517. 31698253 10.1016/j.drugalcdep.2019.06.019 PMC7053256

[jad70054-bib-0057] Yeung, E. W. , J. G. Craggs , and I. R. Gizer . 2017. “Comorbidity of Alcohol Use Disorder and Chronic Pain: Genetic Influences on Brain Reward and Stress Systems.” Alcoholism: Clinical and Experimental Research 41, no. 11: 1831–1848. 29048744 10.1111/acer.13491 PMC5679730

[jad70054-bib-0058] Zellers, S. M. , W. G. Iacono , M. McGue , and S. Vrieze . 2022. “Developmental and Etiological Patterns of Substance Use From Adolescence to Middle Age: A Longitudinal Twin Study.” Drug and Alcohol Dependence 233: 109378. 35248999 10.1016/j.drugalcdep.2022.109378 PMC8957537

