# Supplementary material for: Genetic and Environmental Associations Among Pain, Sleep Disturbances, and Substance Use Intent in Early Adolescence
Source: J Adolesc. 2025 Sep 21;98(1):228–36. doi: 10.1002/jad.70054 (PMC12780654; doi:10.1002/jad.70054)
Supplement: Supplementary file 1 — Supplemental Table 1: ACE Estimates and Unique and Shared Variance Estimates from Full Bivariate Models. [file JAD-98-228-s001.docx]

**Supplemental Table 1. ACE Estimates and Unique and Shared Variance Estimates from Full Bivariate Models**

|  | Common Variance  Estimate (95% CI) | | | Unique Variance  Estimate (95% CI) | | |
| --- | --- | --- | --- | --- | --- | --- |
|  | VCA | VCC | VCE | VUA | VUC | VUE |
| Pain-Sleep | .01 (.00, .05) | **.19 (.03, .39)** | .00 (.00, .01) | **.52 (.29, .71)** | .00 (.00, .18) | **.28 (.20, .37)** |
| Pain-Intent | .04 (.00, .18) | .06 (.00, .15) | .01 (.00, .02) | **.08 (.01, .22)** | **.07 (.01, .15)** | **.75 (.65, .88)** |
| Sleep-Intent | .00 (.00, .01) | .01 (.00, .01) | .00 (.00, .00) | **.53 (.33, .75)** | **.19 (.02, .35)** | **.28 (.21, .36)** |

*Note.* VCA = variance common to additive genetic parameters, VCC = variance common to shared environmental parameters, VCE = variance unique to shared environmental parameters, VUA = variance unique to additive genetic parameters, VUC = variance unique to shared environmental parameters, VCE = variance unique to unique environmental parameters. Significant 95% confidence intervals are bolded.
